# 

**Published:** 1899-01

**Authors:** 


### SELECTIONS.

Article I.—The Practical Value of Chemistry in Dentistry. H. C. Wetmore, D. D. S. .......... 385
" II.—Pyorrhœa Alveolaris—Personal Experience in Treatment. W. Geo. Beers, L. D. S., D. D. S. .......... 394
" III.—Suppurative Inflammation of the Gums and Absorption of the Gums and the Alveolar Process. John W. Riggs, F. A. A. D. S. .......... 401
" IV.—Vigo on Toothache, 1514 .......... 407
" V.—Incipient Decay .......... 411
" VI.—Exposed Pulps .......... 414
" VII.—Treatment of the Cervical Border. Dr Stafford G. Perry .......... 415
" VIII.—Some Properties of Vulcanite Rubber. Dr. S. E. Davenport .......... 419

### COMMUNICATED.

The Chicago Dental Society .......... 421

### MONTHLY SUMMARY.

New Method of Disinfection .......... 422

Cataphoresis—A Warning .......... 423

Transplanting—Resetting .......... 424

Extracts from an Article upon "Some Overlooked Sources of Infection." .......... 425

Tincture of Iodine for the Removal of Deposits .......... 426

Bleeding Gums .......... 426

To Fold Gold Foil Without Contact of the Fingers .......... 427

Matrices .......... 428

Treatment for Exposed Dentin .......... 428

Harden than a Diamond .......... 429

A New Army Hospital .......... 429

Dr. Black's Theory .......... 431

Sun Baths .......... 431

Sterilizing Instruments .......... 431

Soldering Gold Crowns .......... 432

Eucaine .......... 432

Dental Fees .......... 432

